# Supplementary material for: Core-Shell Structured PLGA Particles Having Highly Controllable Ketoprofen Drug Release
Source: Pharmaceutics. 2023 Apr 28;15(5):1355. doi: 10.3390/pharmaceutics15051355 (PMC10223903; doi:10.3390/pharmaceutics15051355)
Supplement: Supplementary file 1 [file pharmaceutics-15-01355-s001.zip › pharmaceutics-2320319-supplementary.pdf]

pharmaceutics

Supplementary Materials: Core-Shell-Structured PLGA Particles With Highly-Controllable Ketoprofen Drug Release

Norbert Varga [1,2], Rita Bélteki [1,2], Ádám Juhász [1,2] and Edit Csapó [1,2,*]

1 MTA-SZTE Lendület "Momentum" Noble Metal Nanostructures Research Group, University of Szeged, H-6720 Rerrich B. sqr. 1, Szeged and Hungary
2 Interdisciplinary Excellence Center, Department of Physical Chemistry and Materials Science, University of Szeged, H-6720 Rerrich B. sqr. 1, Szeged, Hungary
* Correspondence: juhaszne.csapo.edit@med.u-szeged.hu; Tel.: (+36(62)544-476); ORCID: 0000-0002-6980-9524

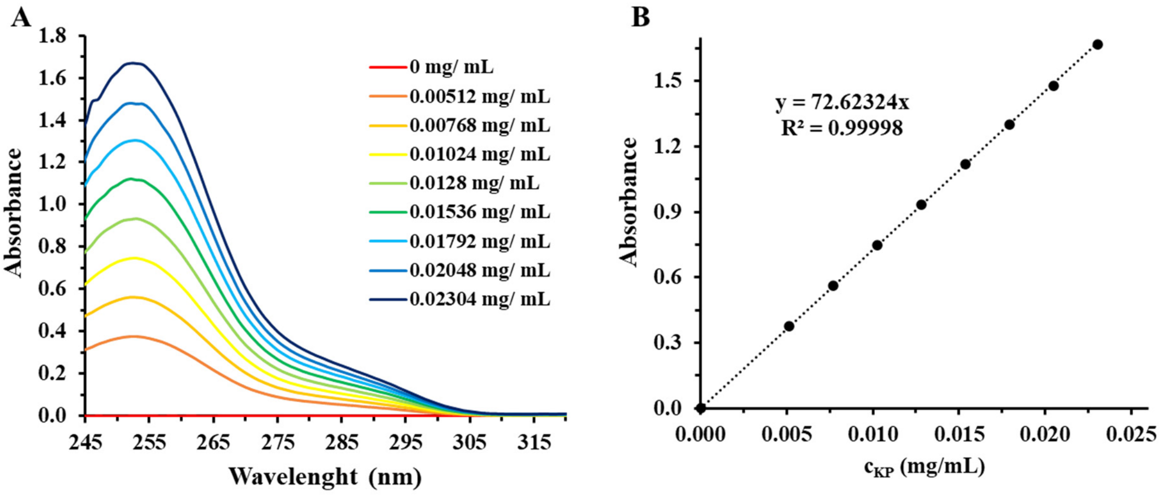

Figure S1. (**A**) UV-Vis spectra and (**B**) calibration curve (at 252 nm) of the ketoprofen in 1,4 – dioxane.

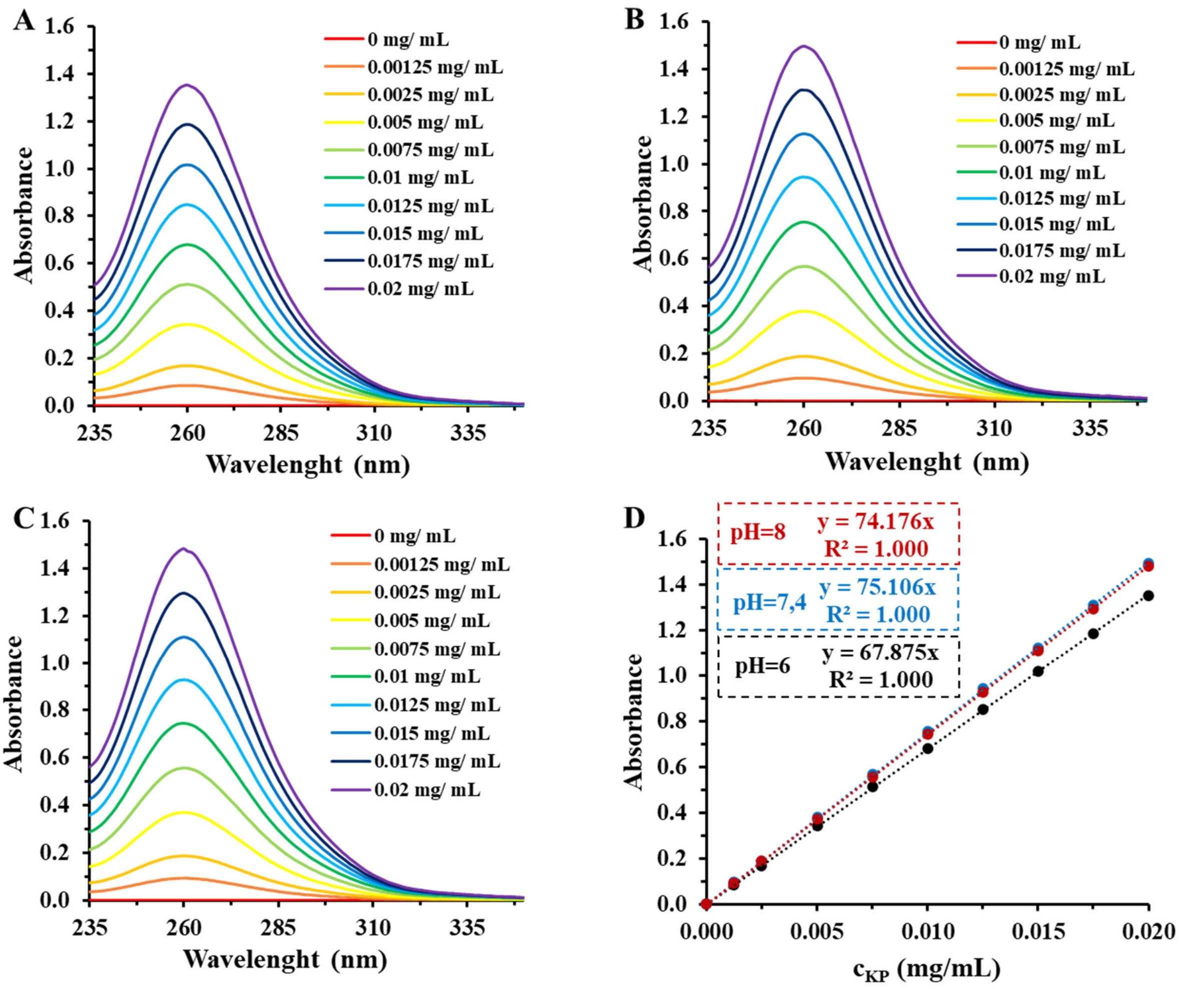

Figure S2. UV-Vis spectra of the ketoprofen at (**A**) pH = 6.0, (**B**) 7.4, and (**C**) 8.0 in phosphate buffer (with 0.9 % NaCl) and (**D**) calibration curve at 260 nm.

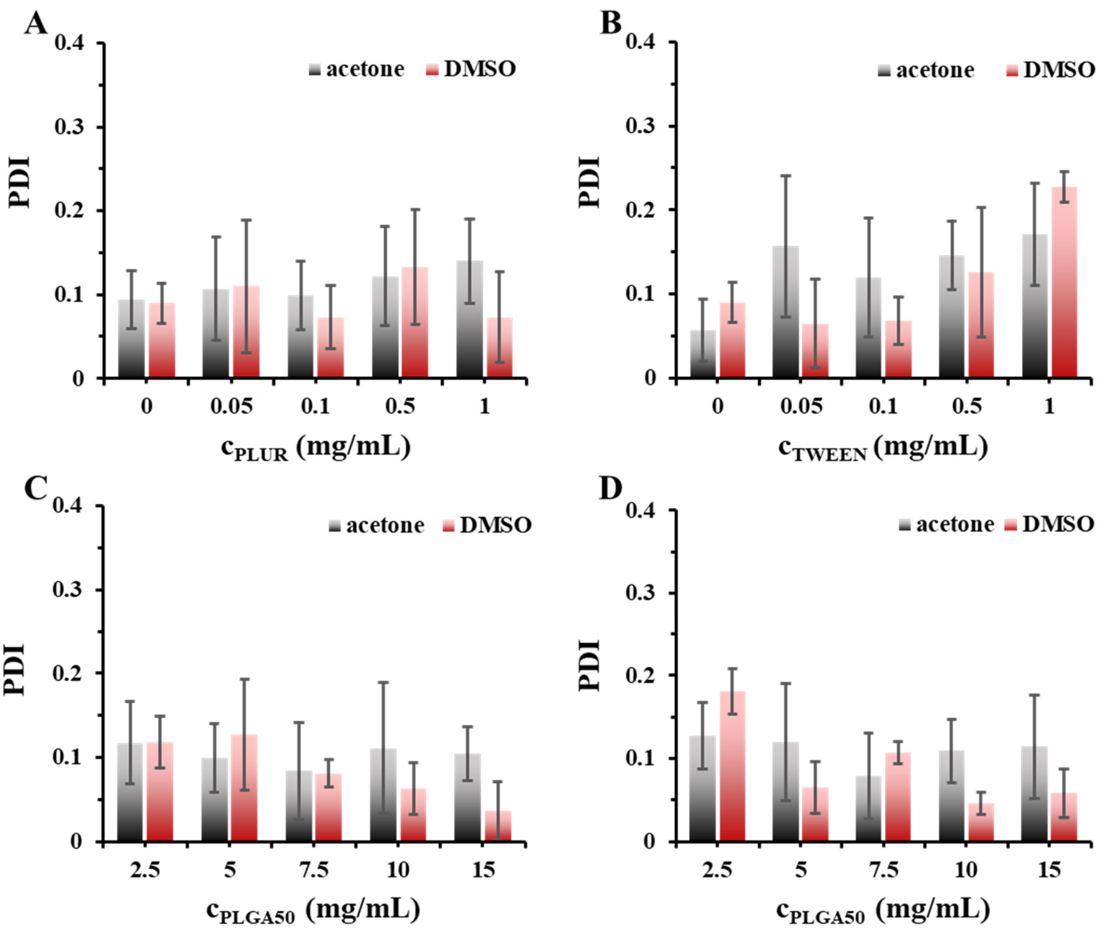

Figure S3. Change of the polydispersity of the drug-free PLGA50 systems at different initial (**A,B**) stabilizer (c_{PLGA50} = 5 mg/mL; $c_{stabilizer}$= 0 - 1 mg/mL; $V_{stabilizer}$ = 5 mL; 1:10 organic solvent:water ratio) and (**C,D**) PLGA (c_{PLGA50} = 2.5 – 15 mg/mL; $c_{stabilizer}$ = 0.1 mg/mL; $V_{stabilizer}$ = 5 mL; 1:10 organic solvent:water ratio) concentrations (**A,C**: PLUR stabilizer; **B,D**: TWEEN stabilizer; acetone and DMSO are marked with black and red column, respectively, in the online form).

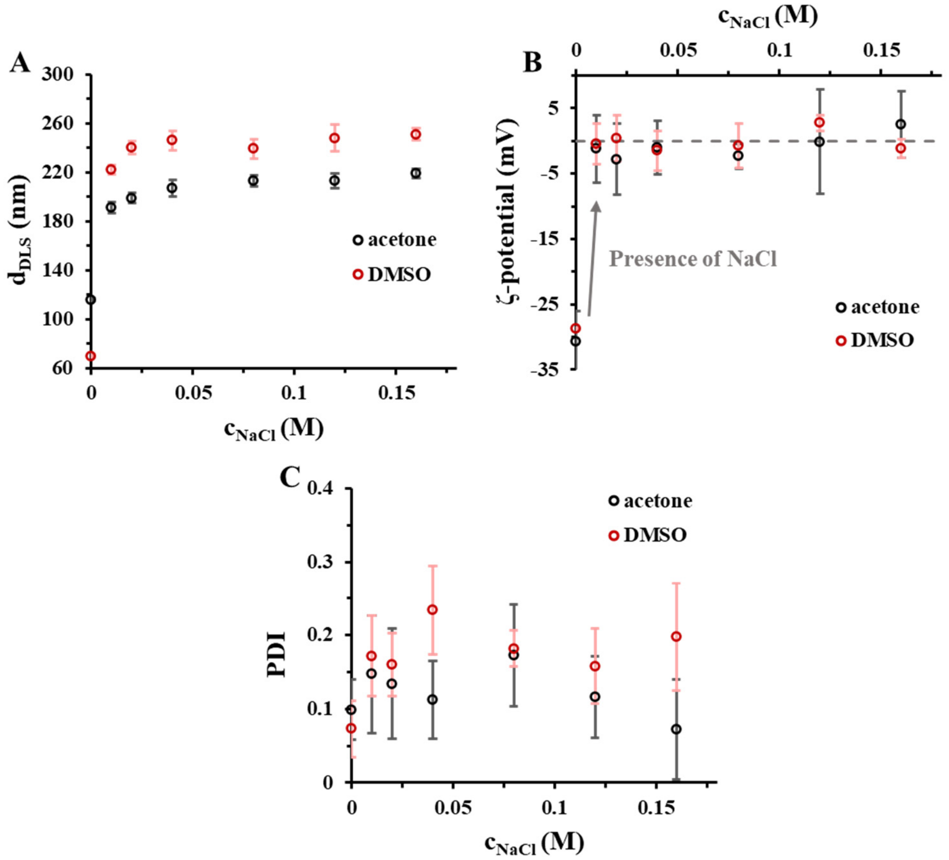

Figure S4. (**A**) Hydrodynamic diameter, (**B**) ζ-potential, and (**C**) polydispersity (PDI) of the drug-free PLUR/PLGA50 colloidal particles at different NaCl concentrations (c_{PLGA50} = 5 mg/mL; $c_{stabilizer}$= 0.1 mg/mL; $V_{stabilizer}$= 5 mL; 1:10 organic solvent:water ratio; acetone and DMSO are marked with black and red ○ symbol, respectivelly, in the online form).

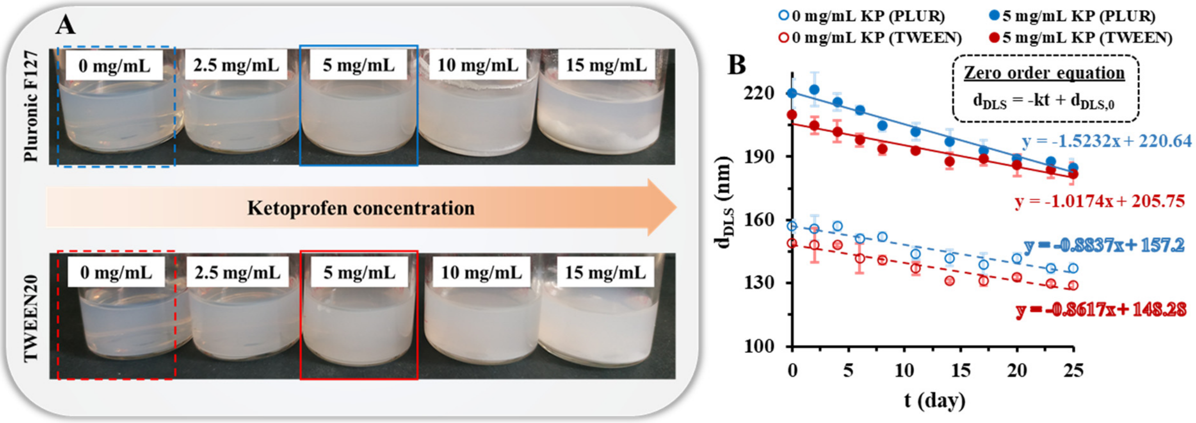

Figure S5. (A) Representative pictures from the prepared KP-loaded PLGA50 NPs at different drug concentrations and (**B**) change of the hydrodynamic diameters of the drug-free and 5 mg/mL KP-contained carriers in 0–25 day range, with the fitted zero order equation (c_{PLGA50} = 10 mg/mL; $c_{PLUR/TWEEN}$ = 0.1 mg/mL, $V_{PLUR/TWEEN}$ = 5 mL; 1:10 organic solvent:water ratio).

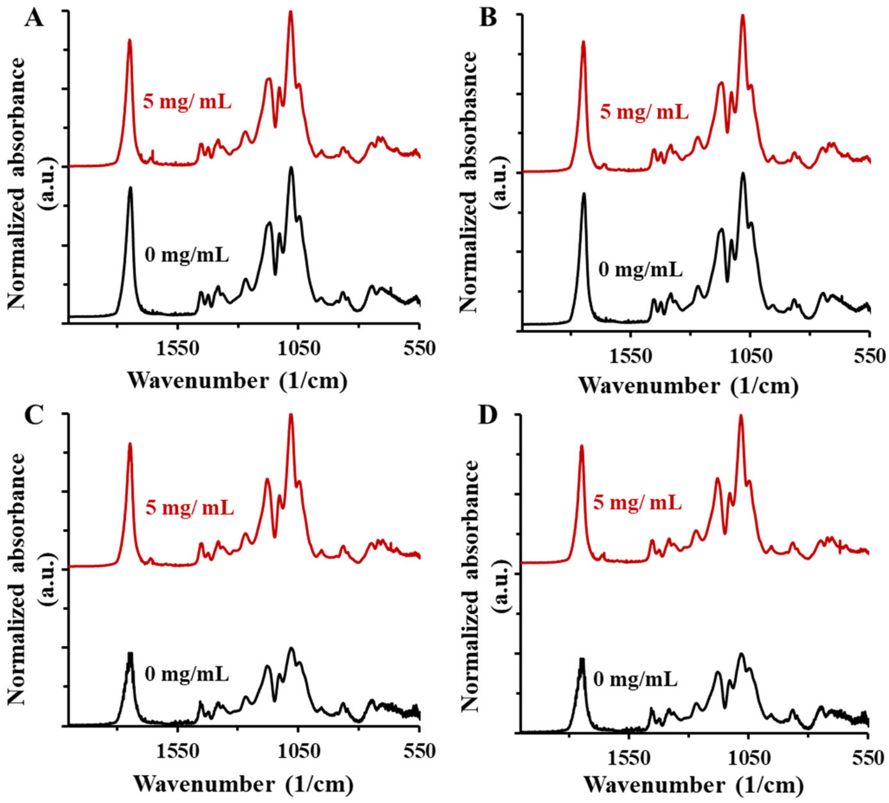

Figure S6. FT-IR spectra of the KP-loaded (**A,B**) PLGA65 and (**C,D**) 75 NPs (c_{PLGA50} = 10 mg/mL; c_{KP} = 0 and 5 mg/mL, $c_{PLUR/TWEEN}$ = 0.1 mg/mL; **A,C**: PLUR stabilizer; **B,D**: TWEEN stabilizer).

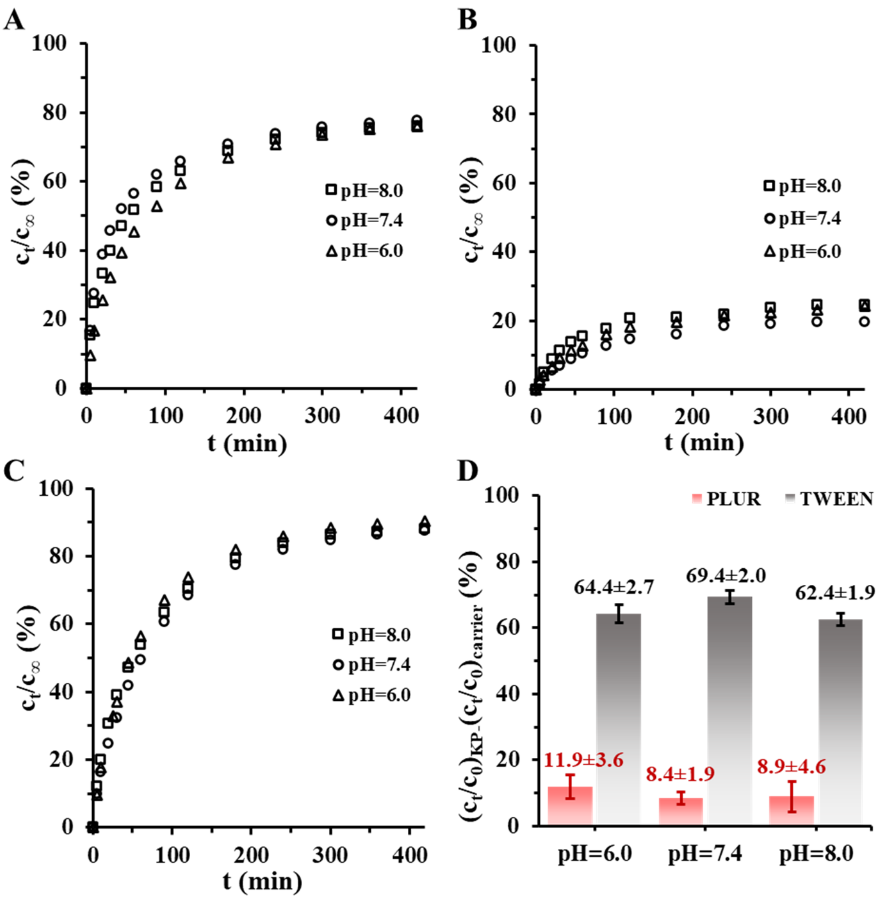

Figure S7. The dissolution curves of the (**A**) PLUR/PLGA50/KP NPs, (**B**) TWEEN/PLGA50/KP NPs, and (**C**) solid KP at different pH levels (T = 37 °C; pH = 6 - 8 phosphate buffer medium; 0.9 % NaCl), and (**D**) the retention of the PLGA50/KP NPs, corrected with released drug values of the solid KP at 420 min.

Table S1. The hydrodynamic diameter (d_{DLS}), polydispersity (PDI), encapsulation efficiency (EE (%)), and drug-loading (DL (%)) of the KP-loaded PLGA NPs (c_{PLGA} = 10 mg/mL; V_{PLGA50} = 0.5 mL; $c_{PLUR/TWEEN}$ = 0.1 mg/mL; $V_{PLUR/TWEEN}$ = 5 mL).

		c_{KP} (mg/mL)	d_{DLS} ± SD (nm)	PDI ± SD	EE ± SD (%)	DL ± SD (%)
PLURONIC F127	PLGA50	0.0	149±3	0.144±0.054	-	-
		5.0	212±6	0.106±0.074	18.2±1.4	8.3±0.6
	PLGA65	0.0	160±2	0.150±0.046	-	-
		5.0	226±3	0.121±0.018	14.9±2.1	6.9±0.9
	PLGA75	0.0	162±3	0.116±0.071	-	-
		5.0	232±6	0.060±0.043	16.5±0.6	7.6±0.3
TWEEN 20	PLGA50	0.0	139±2	0.076±0.030	-	-
		5.0	198±4	0.131±0.057	14.3±1.1	6.7±0.5
	PLGA65	0.0	161±3	0.067±0.034	-	-
		5.0	233±2	0.137±0.084	14.6±2.5	6.8±1.1
	PLGA75	0.0	158±2	0.120±0.065	-	-
		5.0	227±8	0.087±0.020	16.2±3.8	7.5±1.6

Table S2. The determined parameters of KP dissolution after fitting several kinetic formulations.

	Formulation	Second Order		Weibull			Korsmeyer–Peppas		
		k ($mLmg^{-1}s^{-1}$)	R^2	a	b	R^2	k_m (s^{-n})	n	R^2
	solid KP	0.0205	0.9982	0.0025	0.6834	0.9953	0.0207	0.3789	0.9642
PLUR	PLGA50	0.0503	0.9767	0.0228	0.4348	0.9830	0.0682	0.2503	0.9551
	PLGA65	0.0585	0.9918	0.0112	0.5247	0.9769	0.0533	0.2811	0.9346
	PLGA75	0.0909	0.9916	0.0175	0.5006	0.9764	0.0860	0.2397	0.9291
TWEEN	PLGA50	0.0019	0.8420	0.0031	0.4190	0.9742	0.0037	0.3915	0.9714
	PLGA65	0.0041	0.9000	0.0039	0.4645	0.9836	0.0058	0.4059	0.9789
	PLGA75	0.0198	0.9569	0.0076	0.4957	0.9829	0.0179	0.3632	0.9683
